# Ruptured Emphysematous Liver Abscess: An Unusual Presentation in Kochs

**DOI:** 10.3390/clinpract11020029

**Published:** 2021-04-02

**Authors:** Girish D. Bakhshi, Gurpreet Singh, Jessica Shah, Dinesh Pawar, Srinivas Ram, Nimisha Raut

**Affiliations:** Department of Surgery, Grant Govt Medical College & Sir J.J. Group of Hospitals, Mumbai 400008, India; gdbakhshi@gmail.com (G.D.B.); jess27.js@gmail.com (J.S.); dinesh.pawartoo750@gmail.com (D.P.); srinivas1990seena@gmail.com (S.R.); nimnightingale@gmail.com (N.R.)

**Keywords:** gas forming liver abscess, rupture, tuberculosis

## Abstract

Gas forming liver abscess (GFLA) though rare is seen in diabetic patients. Rupture of such abscesses usually requires surgical intervention. These cases are associated with high morbidity and mortality due to sepsis. Tuberculous liver abscesses are more often silent in presentation. GFLA formed in the background of a tuberculous liver abscess is rare. We present a case of ruptured GFLA with underlying tuberculous pathology in a normoglycemic patient. The abscess was managed by image guided intervention. A brief case report along with review of literature is presented.

## 1. Introduction

Emphysematous liver abscess was initially described by Smith [1] in 1944. It is reported to have the highest incidence in South East Asia especially Taiwan [2]. Emphysematous liver abscess have an incidence ranging from 6–24% and is lethal in 25% of patients. This form of liver abscess has emerged with higher rates of distant complications and poor prognosis. It is often caused by *klebsiella pneumonia* and seen commonly in patients with diabetes mellitus. Spontaneous rupture of gas forming liver abscess (GFLA) is seen in almost 5.4% of all liver abscesses [3] and they occur mostly, due to high internal pressure and hepatocellular damage [2]. Tuberculous liver abscess on the other hand is not that rare and usually presents with an indolent course but is never associated with gas formation.

## 2. Case Report

We report a case of a 45-years-old female who was presented to the emergency department with pain in right upper abdomen for 1 month, bilateral pedal edema for 7 days. However, no history of fever or obstruction was found. On examination, she was afebrile with pulse of 90 beats per minute and 98% oxygen saturation on room air. Per abdomen examination revealed tenderness in the right hypochondrium with localized guarding. There was no rigidity and bowel sounds were present. Hematological and biochemical investigations were suggestive of inflammatory response with WBC count of 15,800/cu mm and erythrocyte sediment rate (ESR) of 48 mm at the end of 1 hour. She had a *HBA1c* level of 5.8. Her *aspartate transaminase* (AST) level was 371 U/L, alanine aminotransferase (ALT) level was 331 U/L, alkaline phosphatase (ALP) level was 675 U/L, and gamma-glutamyl transpeptidase (γ-GTP) level was 197 mg/dL, indicating liver dysfunction. The patient tested negative for urine ketones and had an arterial blood pH of 7.38 with base deficit of 4.8.

**Figure 1:**
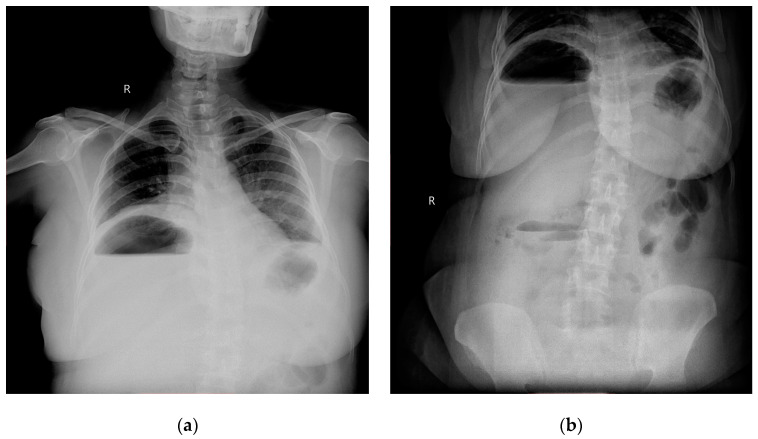
(**a**) Chest roentogram showing air fluid level in the right upper abdomen; (**b**) abdominal radiogram showing air fluid level in right upper with some fluid levels in central abdomen as well.

Radiographic investigations revealed large air fluid level under the right diaphragm with multiple air fluid levels in the right upper part of the abdomen (Figure 1a,b).

**Figure 2:**
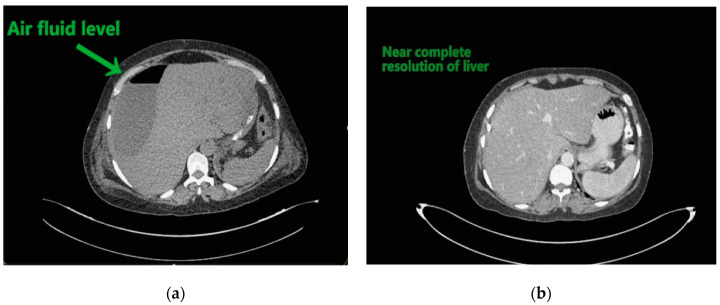
(**a**) Liver abscess showing air fluid levels; (**b**) showing resolution after 10 days.

**Figure 3:**
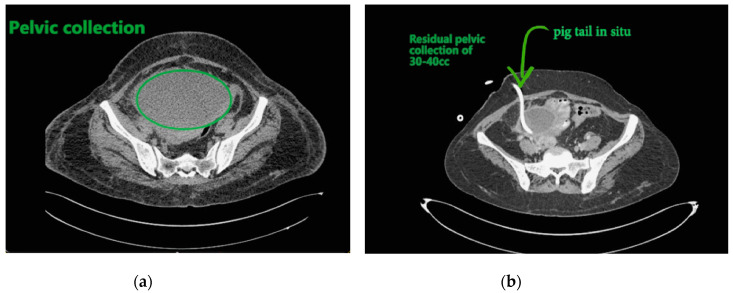
(**a**) Pelvic collection; (**b**) showing residual collection in pelvis with drainage catheter in situ.

Contemplating unusual location of air fluid level in an otherwise stable patient, contrast enhanced computed tomography (CECT) of the abdomen and pelvis was done. CECT was suggestive of liver abscess in segment VII, VI which was 300 cc liquified with gas formation within. The abscess had ruptured with collection tracking along the right paracolic gutter till pelvis with 800 cc liquified large collection noted within (Figure 2a and Figure 3a).

The patient underwent ultrasound guided percutaneous abscess drainage and placement of 12-Fr and 14-Fr catheter in liver and pelvic collections respectively. The aspirate was purulent and *Klebsiella pneumoniae* was detected in cultures. Blood culture was negative even on repeated samples. Gene xpert (CB NAAT) was negative for mycobacterium tuberculosis (MTB). Fluid samples were also negative for malignant cells. She was started on antibiotics *Piperacillin and tazobactum* combination and monitored for blood glucose levels as a high suspicion of diabetes. Antibiotics was switched to *imipenem* as per the sensitivity of fluid cultures. She was tested negative for human immunodeficiency virus (HIV). Conservative management was continued as the patient improved with a repeat CECT after 10 days showing complete resolution of liver abscess and 30–40 cc residual collection in pelvis. Her liver enzymes became normal. Drains were removed on day 12 and day 14 from liver and pelvis respectively. She developed an induration of 13 mm when tested for tuberculin skin antigen test at 72 h post inoculation suggesting positive test. Fluid adenosine deaminase (ADA) levels were found to be 250.5 U/L and patient started on category 1 anti-tubercular treatment. Patient was discharged on day 15 without sequelae.

Follow-up of 6 months showed her to be disease and symptom free. She is advised to take anti-tubercular treatment for 3 more months.

## 3. Discussion

Emphysematous liver abscess is defined as the presence of gas within the abscess of liver, with an incidence of 6–24% [4]; with a higher incidence in South East Asia as compared to the west. Mortality rate ranging from (27–30%) warrants prompt diagnosis and urgent intervention. It is caused by *Klebsiella pneumonia* in 80% of cases; however *E. coli, Salmonella typhi* [5] and *Clostridium perfrigens* [6] are also accused of the same. The virulence factors of *K. pneumoniae* are reported to be rmp A (regulator of mucoid phenotype gene A), mag A (mucoid-associated gene A), capsular serotype, lipopolysaccharide, hypermucoviscosity phenotype. Capsular serotypes are more virulent and affects immunocompetent hosts. Chien Chuang et al. showed six virulent capsular types which were more prevalent in the non-DM than the DM (diabetes mellitus) group [7].

Gas production is attributed to the hyperglycemia seen in most of the cases. It is assumed that these facultative anaerobes can grow in anoxic environments by degrading glucose, especially under hyperglycemic conditions. During this process, carbon dioxide is produced and emphysematous liver abscesses are formed. Therefore, this condition is considered to be common in patients with poorly controlled DM [5,8]. However, present case was a 45 year normoglycemic patient. Chang Jae Lee et al. [9] reported 6/25 patients with GFLA associated with normal glycemic indices; mechanism of gas generation in non-diabetics is not yet clear. 

Clinically, ruptured GFLA presents as septic shock or sometimes asymptomatic in diabetic patients due to blunted immunological response. Present case was non-diabetic with localized symptoms and signs with no systemic manifestations [7]. 

Abdominal ultrasound, simple abdominal radiography, and other imaging techniques are useful for diagnosis, but CT is the best method for sensitive detection of gas within abscesses. In the present case X-ray showed air under right dome of Diaphragm and as patient was clinically stable, CECT was done which confirmed the diagnosis.

Most common complication in GFLA is respiratory complication with *septic embolisation*; *septic shock, rupture, endophthalmitis,* and *meningitis* are also seen [10]. Chou et al. verified that rupture can occur as a result of strong tissue damage and rise in internal pressure due to gas formation [11]. The fatality rate (27–30%) is higher than that of non-gas forming liver abscess (2–12%) and is often associated with features of peritonitis and required emergent surgical intervention. Present case has an indolent nature of disease with a contained rupture tracking only to the right paracolic gutter walling off in pelvis with no signs of peritonitis. Hence ultrasound guided percutaneous drainage of both liver abscess and pelvic collection was done. Pus fluid was sent for analysis of adenosine deaminase (ADA) as this patient did not show features of peritonitis as seen in other ruptured abscesses. This abscess developed gradually over a period of 1 month as per the duration of symptoms. It also allowed the body to wall off the ruptured abscess resulting in localized signs.

Fluid ADA level above 33 U/L is sensitive to 83.6% and 92.7% specific in population-based studies in diagnosis of *mycobacterium Tuberculosis* MTB [12]. Present patient had ADA levels of 250.53 U/L and considering afebrile, torpid course raised high suspicion of tuberculosis. Hence patient was started on category 1 anti-tubercular treatment to which he responded well. This also compels us to think that a tuberculous liver abscess which is asymptomatic to start with can get secondarily infected. This secondary infection results in symptoms and signs, and rarely GFLA.

## 4. Conclusions

Emphysematous liver abscess in a normoglycemic patient, with indolent course and contained rupture has instigated quest for new pathways in pathogenesis and shift toward conservative management in such cases. Besides treatment of acute infection, tuberculous work up should be considered in patients with indolent course. However, larger studies will be required to substantiate the results.

## Data Availability

All data are included in the manuscript.
